# How does chronic pain lead to memory loss?

**DOI:** 10.7554/eLife.105633

**Published:** 2025-01-27

**Authors:** Suelen Pereira, Ivan Tomsic, Robson da Costa, Mychael V Lourenco

**Affiliations:** 1 School of Pharmacy, Federal University of Rio de Janeiro Rio de Janeiro Brazil; 2 Institute of Medical Biochemistry Leopoldo de Meis, Federal University of Rio de Janeiro Rio de Janeiro Brazil; 3 Department of Biochemistry and Tissue Biology, Institute of Biology, State University of Campinas Campinas Brazil

**Keywords:** chronic pain, memory, dentate gyrus, sphingosine 1-phosphate, synaptic plasticity, Mouse

## Abstract

A dysfunctional signaling pathway in the hippocampus has been linked to chronic pain-related memory impairment in mice.

**Related research article** Cui M, Pan X, Fan Z, Wu S, Ji R, Wang X, Kong X, Wu Z, Song L, Song W, Yang JX, Zhang H, Zhang H, Ding HL, Cao JL. 2024. Dysfunctional S1P/S1PR1 signaling in the dentate gyrus drives vulnerability of chronic pain-related memory impairment. *eLife* **13**:RP99862. doi: 10.7554/eLife.99862.

Around 30% of the world’s population have the misfortune to suffer from chronic pain. Moreover, about two-thirds of these individuals also experience memory deficits (Liu, 2022). This happens because pain and memory share neural circuits that can mutually influence each other. In particular, humans rely on a range of cognitive processes – such as learning, the recall of past experiences, and decision-making – to interpret painful stimuli and respond to them effectively (Phelps et al., 2021). Therefore, understanding the effects of pain on cognition, especially its effect on memory, is an essential prerequisite for improving the symptoms of these individuals and enhancing their quality of life.

The hippocampus, a region of the brain that is critical to various aspects of memory, is often affected in individuals experiencing chronic pain. Previous studies have shown that chronic neuropathic pain impairs spatial memory, and also suppresses the production of new neurons in the hippocampus, which may explain the memory deficits observed in some individuals with chronic pain (Xia et al., 2020). However, the underlying mechanisms have remained largely unclear.

Now, in eLife, Hai-Lei Ding, Jun-Li Cao and colleagues – including Mengqiao Cui, Xiaoyuan Pan and Zhijie Fan (all of Xuzhou Medical University) as joint first authors – report the results of experiments on mice that may help shed light on the link between chronic pain and memory loss (Cui et al., 2024). Like humans, some of the mice seemed more susceptible to the cognitive impairment caused by pain than others. By analyzing these differences, the researchers identified the S1P/S1PR1 signaling pathway as a critical factor influencing this susceptibility. S1PR1, which is short for sphingosine 1-phosphate receptor 1, is a transmembrane receptor protein for a signaling molecule called S1P.

Imagine your brain as a pool of cells communicating through a complex network of synaptic connections that constantly reconfigure to create and store memories. Structures called dendritic spines have an important role in the formation of these connections. Cui et al. report that, in some subjects, chronic pain causes a reduction in the levels of the receptor S1PR1, which is crucial for maintaining the actin cytoskeleton found inside the dendritic spines (Figure 1). As a result of this imbalance, the synaptic connections between the cells weaken, impairing the ability of the brain to recall important information.

**Figure 1.**
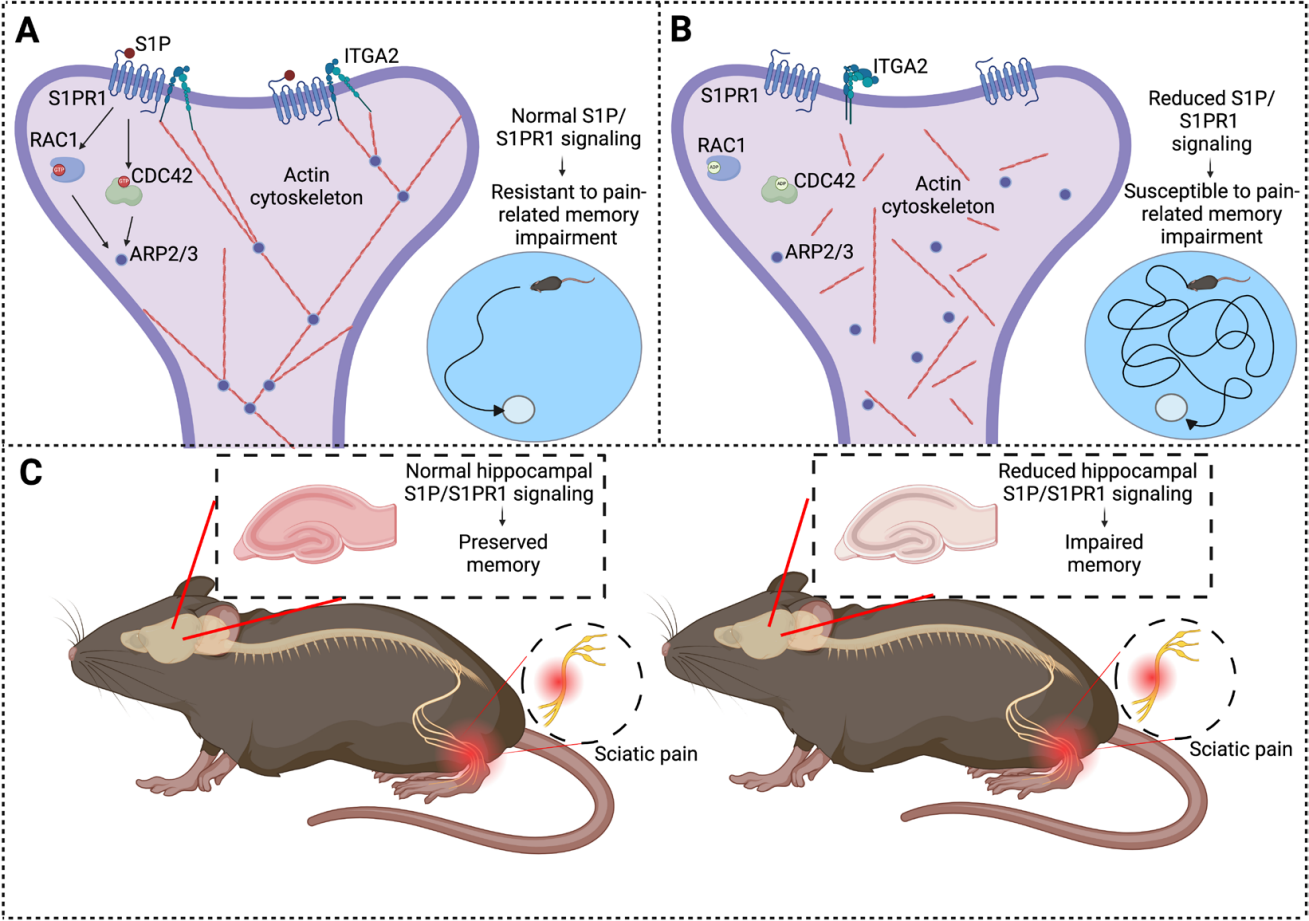
Chronic pain and memory impairment. (**A**) Schematic representation of synaptic signaling in a dendritic spine in the hippocampus under normal conditions. Activation of the S1PR1 receptor by the signaling molecule S1P has an important role in maintaining the actin cytoskeleton (via a number of downstream effectors: RAC1, CDC42, and ARP2/3), as does the interaction between S1PR1 and a protein called ITGA2. An organized actin cytoskeleton (shown in red) supports the structural integrity of the dendritic spine (and also the synapse it forms with an axon (not shown)), and thus has a role in memory preservation. Mice with normal S1P/S1PR1 signaling are resistant to pain-related memory impairment (as evidenced by their performance in memory tests; bottom right). (**B**) Under conditions of reduced S1P/S1PR1 signaling, the structural integrity of the actin cytoskeleton is impaired, leading to synaptic dysfunction. This renders mice susceptible to pain-related memory impairment, which affects their performance in memory tests (bottom right). (**C**) Mouse model illustrating the impact of pain on S1P/S1PR1 signaling in the hippocampus and memory. In the presence of intact signaling (left), hippocampal function and memory are preserved despite sciatic pain. However, reduced hippocampal S1P/S1PR1 signaling correlates with impaired memory in pain conditions (right). ITGA2: integrin alpha-2; S1P/S1PR1; sphingosine 1-phosphate/sphingosine 1-phosphate receptor 1. Created with BioRender.com.

To draw these conclusions, Cui et al. – who are based at Xuzhou Medical University and the University of Macau – used a mouse model in which chronic neuropathic pain was induced via a chronic constriction injury of the sciatic nerve in the left hind paw. The animals were then subjected to tests to assess two types of chronic pain – mechanical allodynia and thermal hyperalgesia (which are characterized by excessive sensitivity to touch and heat respectively). The researchers also subjected the mice to two behavioral tests – using a Y-shaped maze and a Morris water maze – to assess learning and spatial memory.

Based on these assessments, mice were split into two groups – those susceptible to memory impairment, and those resistant to memory impairment. RNA sequencing and gain/loss-of-function studies revealed that the S1P/S1PR1 signaling pathway plays a key role in determining vulnerability to pain-related cognitive impairment. Additionally, susceptible mice exhibited signs of reduced excitatory synapse formation and changes in dendritic spine morphology in the hippocampus. Together, these findings suggest that when S1PR1 was switched off in the hippocampus, synaptic structure was compromised. Building on this concept, enhancing S1PR1 activity through genetic and pharmacological approaches significantly improved memory performance in the mice. These results suggest that S1PR1 could be a promising therapeutic target for preventing the memory loss associated with chronic pain.

The study also revealed that defective S1P/S1PR1 signaling in susceptible mice disrupted the actin cytoskeleton, which is essential for the stability and adaptability of the synaptic connections in the brain (Alimohamadi et al., 2021). The organization of the actin cytoskeleton depends on a process called actin polymerization, which relies on interactions between S1PR1 and a protein called ITGA2: however, these interactions are impaired when S1P/S1PR1 signaling is defective. The disruption of the actin cytoskeleton weakens synapses and impairs the formation of new connections, which are vital for learning and memory. The imbalance in this signaling pathway provides insights into how chronic pain impacts cognitive function.

By identifying S1PR1 as a central element in this interaction, Cui et al. have paved the way for innovative therapeutic strategies, moving beyond mere pain relief to potentially protect and restore impaired cognitive functions. With potential applications for disorders such as arthritis, fibromyalgia and migraine, this research indicates a paradigm shift toward precision treatments that address both the physical and cognitive dimensions of chronic pain.

While these findings are promising, many questions remain. For example, can S1PR1 signaling influence other types of memory beyond those studied here? Could these mechanisms also be relevant in other neurological conditions involving pain and cognitive dysfunction? Additionally, how well do these results in mice translate to humans?

Addressing these questions will be vital if we are to develop treatments that can help individuals who suffer from both chronic pain and memory loss.
